# Vitamins in the Pathogenesis of Prostate Cancer: Implications for Prevention and Therapeutic Support

**DOI:** 10.3390/ijms26094336

**Published:** 2025-05-02

**Authors:** Kinga Królikowska, Jakub Kiślak, Karolina Orywal, Monika Zajkowska

**Affiliations:** 1 Department of Population Medicine and Lifestyle Diseases Prevention, The Faculty of Medicine, Medical University of Białystok, 15-269 Białystok, Poland; 2 Department of Biochemical Diagnostics, Medical University of Bialystok, Waszyngtona 15A St., 15-269 Bialystok, Poland; karolina.orywal@umb.edu.pl; 3 Department of Neurodegeneration Diagnostics, Medical University of Bialystok, Waszyngtona 15A St., 15-269 Bialystok, Poland

**Keywords:** healthy diet, nutrients, food, cancer therapy

## Abstract

Prostate cancer is one of the most common malignancies in men and represents a major challenge for modern medicine. In recent years, there has been growing interest in the role of vitamins in the pathogenesis of this disease, as well as their potential impact on prevention and supportive treatment. Studies conducted so far suggest that certain vitamins may exhibit antioxidant and anti-inflammatory properties, thus supporting the immune response, which may influence cancer risk and treatment efficacy. This article aims to present the current state of knowledge on the role of vitamins in prostate cancer, focusing on their potential importance both in prevention and as an adjunct to therapy. Vitamins A, D, E, C, and B-group compounds may influence prostate cancer development and progression through mechanisms such as antioxidant activity, regulation of cell proliferation, apoptosis, and immune modulation. Despite promising insights from basic research, clinical studies remain inconclusive, and the effects of vitamin supplementation depend on factors like dosage, form, and individual variability. Therefore, a balanced diet rich in natural vitamins is recommended, while further research is needed to clarify their therapeutic and preventive roles in prostate cancer.

## 1. Introduction

Prostate cancer is one of the most commonly diagnosed malignancies in men worldwide. Data available at the Global Cancer Observatory (GCO; GLOBOCAN) shows that there were approximately 1.4 million new cases of prostate cancer in 2022, accounting for 15 percent of all cancer cases in men and thus making it the second most common cause of cancer-related death in this group [1]. Incidence rates vary by geographic region, with the highest incidence in developed countries, suggesting a significant influence of both genetic and environmental factors [2]. In Europe, prostate cancer represents the most frequently diagnosed malignancy among men, accounting for over 20% of all cancers within this demographic. The highest incidence rates are observed in Western and Northern European nations, including Sweden, Norway, Ireland, and France. These elevated rates are largely attributable to the widespread implementation of prostate-specific antigen (PSA) screening protocols and the presence of highly developed healthcare systems, which facilitate early detection and diagnosis [3]. Despite these high incidence figures, mortality rates in Europe remain comparatively low, particularly in countries with advanced oncological care, owing to early intervention strategies and the availability of modern therapeutic modalities [4]. In contrast, African countries exhibit relatively lower incidence rates of prostate cancer. However, mortality rates in this continent are among the highest globally. This discrepancy is primarily driven by late detection of the disease, limited access to screening and comprehensive treatment options, and generally low levels of health awareness among the male population. Consequently, prostate cancers are frequently diagnosed at advanced clinical stages, significantly impairing prognosis and survival outcomes [3,5]. In Asia, incidence rates are generally lower, particularly in Southeast Asia, but there are increasing numbers of cases in developing countries such as China and India [3,4]. This trend is attributed to changes in lifestyle, urbanization and increased availability of diagnostic tests. Despite the lower incidence compared to the West Asia, the increase in prostate cancer detection in Asia is becoming visible [4]. In South America, prostate cancer is the most prevalent malignancy among men and constitutes a leading cause of cancer-related mortality within this group. Countries including Brazil, Colombia, and Argentina report relatively high incidence and mortality rates, which may be influenced by the coexistence of genetic predispositions, environmental factors, and barriers to accessing effective oncological care [3,4].

Several nations in Africa, Asia, Latin America/Caribbean, and Central and Eastern Europe have reported rising trends. This could be attributable to higher detection rates (incidence) and limited access or availability of therapies (mortality only). These findings emphasize the necessity of improving access to early detection and treatment services in order to lower the large illness burden in these regions [1]. The development of prostate cancer results from a multifactorial pathogenetic process in which the interaction between genetic predisposition, hormonal and environmental factors plays a significant role. Androgens, especially testosterone and its active metabolite dihydrotestosterone (DHT), stimulate tumor cell proliferation through activation of androgen receptors [6]. Chronic inflammation and oxidative stress are also important in the pathogenesis of this disease, leading to DNA damage and genomic instability, favoring the initiation of neoplastic processes [7]. Not only genetic, but also environmental determinants are responsible for these processes. Mutations in the BRCA1 and BRCA2 genes increase the risk of developing aggressive forms of prostate cancer. Lifestyle also plays an important role, as a diet high in saturated fat and red meat combined with a low content of fiber, vegetables and fruits has been linked to a higher risk of this disease. In contrast, consumption of foods rich in antioxidants, such as lycopene (present in tomatoes) and omega-3 fatty acids, may have a protective effect [8,9]. Additionally, exposure to chemicals, including pesticides, heavy metals and air pollutants, may increase the risk of developing prostate cancer. Vitamins are essential micronutrients that play a crucial role in maintaining homeostasis. Their influence on cancer pathophysiology has become a focal point of extensive scientific investigation, as researchers seek to elucidate the mechanisms by which these compounds may affect tumorigenesis and cancer progression [10]. In the context of prostate cancer, attention is drawn to their antioxidant and immunomodulatory effects and the regulation of cell division [11]. Some vitamins may promote DNA repair mechanisms, which reduce the risk of mutations leading to neoplastic transformation [12]. Others, on the other hand, can inhibit tumor cell proliferation and induce apoptosis, which can limit cancer cells growth. Vitamins can be divided into two main groups: fat-soluble and water-soluble. The first group comprises fat-soluble vitamins, which can be stored within the adipose tissue of the body. This property enables their prolonged retention and availability for physiological functions over extended periods [13]. Water-soluble vitamins, alternatively, are not stored in substantial quantities within the body. Consequently, a consistent dietary intake of these vitamins is crucial for maintaining optimal physiological function [14]. Both groups of vitamins play a significant role in maintaining oxidative balance, regulating the cell cycle and supporting immune system function, making them important factors in both prevention as well as cancer therapy. Table 1 provides a comprehensive overview of vitamins, detailing their physiological roles and their occurrence within the human body.

Epidemiological studies suggest that a diet rich in vitamins with antitumor properties may have an effect of reducing the risk of prostate cancer and the aggressiveness of the disease in patients who have already been diagnosed with cancer. In addition, specific vitamins have been shown to modulate the immune response, thereby enhancing the body’s capacity against cancer cells [15]. The effect of vitamins on hormonal metabolism is another important element in the prevention and treatment of prostate cancer, especially in the context of regulating androgen activity [16]. This paper aimed to conduct a comprehensive analysis of the existing scientific literature regarding the role of vitamins in the pathogenesis of prostate cancer, to assess their significance in preventive strategies, and to evaluate their potential influence on the efficacy of cancer therapies.

## 2. Methods

**Figure 1:**
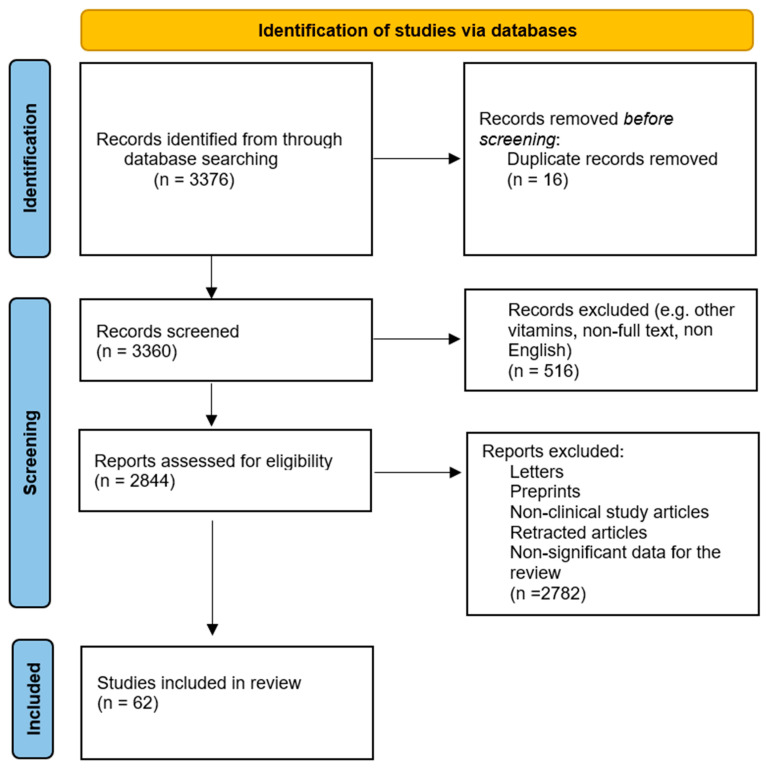
Prisma 2020 flow diagram depicting the methods for studies included in the review.

We have performed a comprehensive literature search in the MEDLAB/Pubmed electronic database using the keyword “vitamin and prostate cancer” (*n* = 3376). The next step involved removing duplicates and choosing vitamins essential for the review: “Vitamin D and prostate cancer” (*n* = 1316), “Vitamin A and prostate cancer” (*n* = 289), “Vitamin E and prostate cancer” (*n* = 615), “Vitamins B and prostate cancer” (*n* = 470), “Vitamin C and prostate cancer” (*n* = 235). Then only publications in English, and with full-text available, were selected. In the next step, letters, pre-prints, non-clinical study articles, retracted articles and non-significant data for the review were excluded (Figure 1).

## 3. Role of Fat-Soluble Vitamins in Prostate Cancer

### 3.1. Vitamin D

In recent years, there has been an increased focus on the role of vitamin D in the prevention and advancement of prostate cancer. Known primarily for its regulation of calcium-phosphate metabolism, vitamin D also exhibits immunomodulatory and antiproliferative properties, making it a potential protective factor in oncology [17]. Vitamin D occurs in the body in form of cholecalciferol (D_3_) and ergocalciferol (D_2_), which are metabolized in the liver to 25-hydroxyvitamin D (25(OH)D) and then in kidneys and other tissues, including the prostate, to the active form—1,25-dihydroxyvitamin D (1,25(OH)_2_D), or calcitriol [18]. Calcitriol acts mainly through the vitamin D receptor (VDR), which, when activated, regulates the expression of various genes related to cell proliferation, apoptosis and differentiation [19]. Prostate cancer cells express the VDR, suggesting that vitamin D may play an important role in the pathogenesis and progression of this cancer. Furthermore, the enzyme 1α-hydroxylase (CYP27B1), responsible for the conversion of 25(OH)D to 1,25(OH)_2_D, is also present in prostate cells, signifying that vitamin D may act locally as an autocrine and paracrine factor [20]. Numerous in vitro and in vivo studies indicate that vitamin D may act as an inhibitor of prostate cancer development through different mechanisms. Calcitriol regulates the cell cycle through the expression of genes such as CDKN1A (p21) and CDKN1B (p27), which are inhibitors of cyclin-dependent kinases (CDKs), arresting the cell cycle in the G1 phase [21]. In addition, activation of the VDR can lead to increased expression of proapoptotic Bax protein and downregulation of anti-apoptotic Bcl-2, which promotes apoptosis of cancer cells [22]. Prostate cancer progression also depends on its ability to form new blood vessels, and calcitriol inhibits angiogenesis by reducing the expression of pro-angiogenic factors such as VEGF (vascular endothelial growth factor) and HIF-1α (hypoxia-inducible factor 1-alpha) [23]. Inflammation is one of the factors promoting prostate cancer progression. Vitamin D shows anti-inflammatory properties by inhibiting the production of pro-inflammatory cytokines (e.g., IL-6, IL-8, TNF-α) and increasing the expression of IL-10, which is an anti-inflammatory cytokine [24]. In addition, calcitriol reduces the ability of prostate cancer cells to migrate and invade by regulating of the extracellular matrix metalloproteinases (MMPs) and their inhibitors (TIMPs) expression, thereby reducing basement membrane degradation and tumor infiltration [25].

The results of epidemiological studies on the association between vitamin D levels and prostate cancer risk are inconclusive. Some studies indicate that low blood 25(OH)D levels are associated with a higher risk of aggressive prostate cancer, while other meta-analyses show no clear correlation [26,27]. Clinical trials on vitamin D supplementation in patients with prostate cancer have also produced diverse results. One randomized trial showed that vitamin D supplementation (4000 IU/day) in patients with low-grade prostate cancer reduced the increase in PSA (prostate-specific antigen) levels, suggesting a possible beneficial effect [28]. However, in other studies, high doses of vitamin D showed no significant improvement in patient survival [29].

Vitamin D appears to play an important role in regulating prostate cancer growth and progression through antiproliferative, proapoptotic, anti-inflammatory and anti-angiogenic mechanisms. Despite promising results from basic research, clinical evidence on the efficacy of vitamin D supplementation in the prevention and treatment of prostate cancer remains inconclusive.

### 3.2. Vitamin A and Derivatives

Vitamin A and its derivatives, known as retinoids, play an important role in regulating cell proliferation, differentiation and apoptosis, making them key factors in the context of cancer, including prostate cancer. Studies suggest that retinoids may affect signaling pathways involved in cancer progression, making them a promising therapeutic target. Vitamin A includes compounds such as retinol, retinal and retinoic acid. It is essential for the normal function of epithelial tissues and their metabolites, especially retinoic acid, which regulates gene expression through the activation of nuclear receptors RAR (retinoic acid receptors) and RXR (retinoid X receptors) [30]. The biological effects of retinoids include control of cellular proliferation and induction of apoptosis, which is of key importance in the context of cancers such as prostate cancer. Deregulation of these processes can lead to increased tumor aggressiveness and resistance to therapy.

Studies indicate that vitamin A levels and the activity of its metabolites may influence the progression of prostate cancer. A key mechanism of retinoids action is the regulation of cell cycle- and apoptosis-related gene expression, and all-trans-retinoic acid (ATRA) has been shown to exert anti-proliferative effects by inhibiting cyclin D1 expression and activating pro-apoptotic genes [31]. Additionally, retinoids can reduce the invasiveness of prostate cancer cells by regulating the activity of matrix metalloproteinases (MMPs) and affecting androgen receptor-related (AR) signaling pathways [32].

Due to the ability of retinoids to regulate tumor cell proliferation and differentiation, their potential therapeutic use in the treatment of prostate cancer is being intensively investigated. Experimental studies have shown that ATRA and synthetic retinoids can inhibit prostate cancer progression and increase cell sensitivity to hormone therapy and chemotherapy [33]. Some clinical studies indicate that low serum retinol levels may be correlated with an increased risk of aggressive prostate cancer [34]. However, the results of epidemiological studies are inconclusive and the use of retinoids in therapy requires further research to determine their efficacy and potential side effects.

### 3.3. Vitamin E

Vitamin E, a fat-soluble compound, occurs naturally in eight different chemical forms, divided into four tocopherols and four tocotrienols. These forms include α, β, γ, and δ-tocopherols, as well as α, β, γ, and δ-tocotrienols. The properties of vitamin E include strong antioxidant, anti-inflammatory effects, as well as a significant impact on blood coagulation [35,36].

Both tocopherols and tocotrienols are known for their potent antioxidant properties, defending cells from damage caused by free radicals. Tocotrienols are more effective in protecting cell membranes due to their wide distribution and interaction with radicals. They are considered more efficient in neutralizing peroxyl radicals than α-tocopherol because of their superior positioning within the phospholipid bilayer and stronger interaction with free radicals in the membrane environment [37].

Unlike α-tocopherol, which dominates in serum, γ-tocopherol exhibits stronger anti-inflammatory and antioxidant effects, protecting against lipid damage. Studies suggest its potential role in cancer prevention, particularly prostate cancer, by inhibiting cancer cell proliferation and inducing apoptosis. Epidemiological data indicate that a high intake of γ-tocopherol is associated with a lower risk of advanced prostate cancer. However, high-dose α-tocopherol supplementation may reduce γ-tocopherol levels, potentially diminishing its beneficial effects. While γ-tocopherol is a promising dietary component, further research is needed [38,39].

A large-scale study, SELECT, conducted on nearly 36,000 patients, aimed to determine whether α-tocopherol and selenium supplementation could prevent prostate cancer. Contrary to expectations, no protective effect was observed, and the analysis revealed a 17% increase in prostate cancer risk among those receiving α-tocopherol. Similar findings were obtained in long-term follow-ups after the ATBC study. Possible explanations include the already optimal α-tocopherol levels in participants, eliminating the need for supplementation, or a potential disruption of the balance between different tocopherols, such as γ-tocopherol. It is also possible that some participants had pre-existing precancerous changes, and high doses of α-tocopherol may have an influence on the acceleration of prostate cancer progression. However, subsequent analyses found no correlation between serum tocopherol levels and cancer risk [36,40].

Research on the association between vitamin E and prostate cancer has yielded conflicting results. Some studies suggest a potentially protective role of α-tocopherol, indicating that higher serum levels may reduce the risk of developing the disease. For example, the Third National Health and Nutrition Examination Survey found an inverse correlation between α-tocopherol and prostate cancer risk factors such as testosterone and estrogen levels. Similar results were observed in studies on smokers. On the other hand, high γ-tocopherol levels have been associated with a lower risk of aggressive prostate cancer [40]. The properties of α and γ tocopherols are listed in Table 2.

## 4. Role of Water-Soluble Vitamins in Prostate Cancer

### 4.1. Vitamin B Group

Metabolic processes involving the transfer of one-carbon groups, in which the MTHFR (methylenetetrahydrofolate reductase) enzyme plays a key role, are essential for DNA synthesis, repair, and modification. B vitamins, including folic acid, are crucial in this process and may influence the risk of prostate cancer. Folic acid, as a central component of this pathway, participates in amino acid synthesis and epigenetic modifications [35,40].

The metabolism of folic acid involves its conversion to tetrahydrofolate (THF) and subsequently to 5-methyl-THF, which is essential for methionine synthesis. This process results in the production of S-adenosylmethionine (SAM), a universal methyl group donor used in DNA and histone modifications. Deficiencies in folic acid and other substances involved in one-carbon metabolism, such as vitamins B6 and B12, choline, or methionine, may lead to abnormalities in DNA methylation, increasing the risk of cancer development [35,40].

Studies on the impact of folic acid on prostate cancer risk have yielded conflicting results. Some analyses suggest that low folic acid levels increase the risk of decease from this cancer, while high dietary folate intake has a protective effect. Other studies indicate a link between high levels of folic acid and vitamin B12 and an increased risk of prostate cancer. Interestingly, folic acid supplementation appears to elevate this risk, whereas dietary folates have the opposite effect. This may suggest that the form of folic acid consumed is of critical importance [35,41].

A study conducted in Norway found that the use of folic acid and vitamin B12 may increase the risk of cancer, including prostate cancer, as well as overall mortality. There are concerns that high doses of folic acid may accelerate the growth of existing cancer cells, possibly by weakening the immune system [35,41].

To mitigate the reliance on findings derived exclusively from Norwegian studies, various analyses, including the ProtecT study, indicate a potential association between elevated serum levels of vitamin B12 and an increased risk of prostate cancer. Specifically, circulating holo-haptocorrin, a transport protein for vitamin B12, appears to be correlated with a heightened risk, while transcobalamin may exhibit a protective effect. Furthermore, although prior meta-analyses have suggested a relationship between folic acid levels and prostate cancer risk, no definitive correlation has been established. It is plausible that folic acid may influence the progression of pre-existing cancer rather than its initiation, which could account for the inconsistencies observed in the research findings [42].

One potential explanation for the observed association between vitamin B12 levels and prostate cancer is the hypothesis that the tumor may elevate vitamin B12 concentrations, possibly through the stimulation of haptocorrin production. Another hypothesis posits that vitamin B12 may play a role in epigenetic processes that could contribute to cancer pathogenesis. Nonetheless, evidence supporting this mechanism remains largely speculative at this stage [42].

### 4.2. Vitamin C

Ascorbic acid (vitamin C) is a potent water-soluble antioxidant that has to be supplied by diet, primarily from fruits and vegetables. Studies suggest that it may have a protective effect against cancer by reducing DNA damage and defending cells from harmful carcinogenic substances. Vitamin C intake can come from both food and supplements, but differences in absorption and biological activity may influence its effects [43,44].

It has been found that a daily increase in vitamin C intake by 150 mg was associated with a reduced risk of developing prostate cancer. This effect was more pronounced in case-control studies than in cohort studies. A geographical analysis revealed that the protective effect was statistically significant in the United States, whereas in Europe, only a trend toward risk reduction was observed [44].

A study conducted in Montreal analyzing the relationship between vitamin C intake and prostate cancer found no association between dietary vitamin C intake and prostate cancer risk, regardless of tumor aggressiveness. These findings remained consistent even after accounting for the use of vitamin supplements, including vitamin C and multivitamins [45].

The mechanisms of vitamin C in prostate cancer prevention suggest that it may protect cells from oxidative damage by neutralizing free radicals. Laboratory studies have shown that vitamin C affects cell count reduction and DNA synthesis in androgen-dependent cells. However, despite earlier suggestions of a potential link between vitamin C and prostate cancer, novel studies, including the randomized Physician’s Health Study II, have not confirmed any correlation between vitamin C supplementation and prostate cancer risk [45].

It should be considered that differences in the bioavailability of vitamin C from diet and supplements may influence study outcomes. While research indicates minor differences in vitamin C absorption from various sources, no evidence has been found to suggest that vitamin C supplementation reduces the risk of prostate cancer [45].

**Figure 2:**
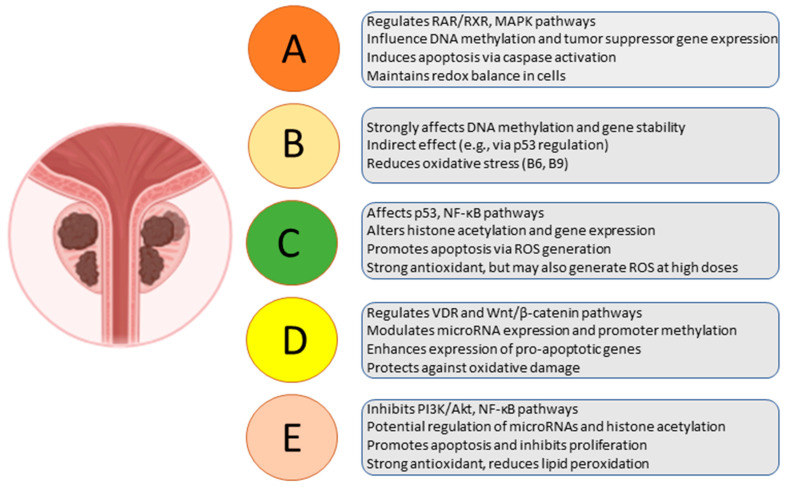
The effects of different vitamins on prostate cancer pathogenesis (signaling pathways, epigenetic effects, apoptosis, and oxidative stress).

A summary of the effects of different vitamins on the pathogenesis of prostate cancer (main mechanisms including signaling pathways, epigenetic modifications, apoptosis, and oxidative stress) is presented in Figure 2. 

## 5. Role of Vitamins in Prostate Cancer Prevention

Prostate cancer is one of the most commonly diagnosed cancers in men, and its prevention represents a significant challenge for modern medicine. Among the factors that may influence its risk reduction, vitamins are of particular interest due to their potential protective effects. The mechanisms by which they may affect cancer prevention include antioxidant properties, regulation of cell proliferation and apoptosis processes and modulation of the immune system [18].

Research conducted so far suggests that adequate levels of certain vitamins may promote protection against prostate cancer, with the efficacy of these vitamins dependent on several factors, such as form of ingestion, dose and interactions with other dietary components. One of the main mechanisms of vitamins’ action is their ability to neutralize free radicals, which can damage the DNA of prostate cells, leading to their neoplastic transformation [46]. Excess free radicals, or oxidative stress, are a factor that promotes genetic mutations and the initiation of the neoplastic process. Vitamins with antioxidant properties can limit this process by stabilizing cellular repair mechanisms and protecting DNA from damage. Another important aspect is the effect of vitamins on the regulation of the cell cycle, which is tightly controlled in a healthy organism. The balance between cell division and programmed death (apoptosis) is crucial, and in the case of tumorigenesis, its disruption leads to uncontrolled cell growth. Certain vitamins can promote apoptosis processes in prostate cells, which in turn can inhibit cancer progression [47]. Vitamins can also modulate the immune response, which promotes defense mechanisms against cancer cells. The immune system plays a key role in identifying and eliminating abnormal cells. However, cancers often develop methods of defense against this response. Certain vitamins can enhance the activity of immune cells, such as T-lymphocytes or macrophages, which promotes the elimination of cancer cells in the early stages of the disease [48]. In addition, vitamins influence the regulation of gene expression associated with cancer development. There is substantial evidence indicating that certain factors may interact with signaling pathways involved in cell growth and differentiation, what could potentially result in a decreased aggressiveness of prostate cancer [49]. By modulating metabolic pathways, vitamins may also affect the process of new blood vessel formation, which is essential for tumor growth and progression. Reducing angiogenesis may result in the inhibition of prostate cancer progression and a reduced risk of metastasis.

Despite numerous studies, results on the effect of vitamin supplementation on prostate cancer risk remain inconclusive. Some studies show benefits from their consumption in adequate amounts as part of a balanced diet, while others suggest that over-supplementation may not have the expected effects and, in some cases, may even increase the risk of tumorigenesis [50]. For example, studies on the use of high doses of certain vitamins, especially synthetic ones, have shown that under certain conditions, they can act pro-oxidative instead of antioxidative. This phenomenon contributes to the promotion of oxidative stress and may potentially elevate the risk of carcinogenesis. Consequently, it is imperative to refrain from excessive consumption of synthetic supplements without prior consultation with a healthcare professional, particularly in the context of long-term supplementation.

It is advisable to provide vitamins primarily through natural sources, such as vegetables, fruits, nuts or vegetable oils, rather than using high doses of supplements. Consuming a diet abundant in vitamins is frequently correlated with the inclusion of various bioactive compounds, such as dietary fiber, polyphenols, and omega-3 fatty acids. These components may synergistically contribute to the body’s protective mechanisms against cancer. Furthermore, evidence suggests that the bioavailability of vitamins derived from natural sources is often superior to that of synthetic alternatives, potentially amplifying their beneficial effects in the context of cancer prevention [51,52].

## 6. Vitamins as Support for Prostate Cancer Therapy

The vitamin D receptor (VDR) plays a crucial role in regulating the growth and differentiation of prostate cancer cells. It has been demonstrated that vitamin D signaling, mediated by VDR, can slow the proliferation of cancer cells, as confirmed by in vitro studies and animal models. The vitamin D receptor (VDR) is expressed in non-epithelial prostate cancer cells and the benign stroma surrounding prostate tumors, suggesting that vitamin D may modulate the prostate tumor microenvironment, particularly in the context of antiandrogen therapy [53].

Blocking androgen activity, such as testosterone and dihydrotestosterone, through antiandrogen therapy is a primary treatment strategy for prostate cancer, especially in advanced cases. However, despite their initial effectiveness, these therapies can eventually lead to treatment resistance, and patients may experience disease recurrence. Consequently, an increasing number of studies focus on the synergistic effects of antiandrogen therapy combined with other approaches, such as vitamin D supplementation. Vitamin D may also influence the tumor microenvironment and inhibit the development of new blood vessels, potentially enhancing the effectiveness of cancer therapies [53,54].

Analyses suggest that vitamin D derivatives may enhance the effects of other anticancer drugs, such as cisplatin and doxorubicin. The combination of calcitriol with these agents may result in reduced progression of prostate cancer. However, the lack of an appropriate calcitriol formulation that allows for the administration of high doses remains a challenge. The new DN-101 formulation offers hope for improved treatment outcomes, and its combination with docetaxel may potentially enhance the response to cancer therapy, nevertheless further research is still needed [54].

By leveraging its antioxidant properties, vitamin C may contribute to improving the quality of life of patients undergoing chemotherapy and radiotherapy, as well as supporting treatment by minimizing therapy-related side effects [55]. Intravenous administration of vitamin C (IV C) in appropriate doses may be a safe and effective method for enhancing antioxidant levels in cancer patients. There is potential synergy between vitamin C and chemotherapy, which could increase treatment efficacy and reduce chemotherapy-related toxicity. The use of vitamin C in oncological therapy has shown promising results, including alleviation of symptoms such as muscle and joint pain and a reduction in inflammation. Studies have demonstrated that injectable vitamin C also supports the stabilization of patients health post-chemotherapy and, in some cases, may contribute to improved treatment response, such as tumor stabilization. There are concerns that the antioxidant effects of vitamin C may interfere with the pro-oxidative mechanisms of chemotherapy. However, the data on this subject remains inconsistent and requires further research [55,56,57].

Vitamin C, especially when administered in intravenous form, has been reported to potentially mitigate the adverse effects associated with chemotherapy and enhance the overall quality of life for patients undergoing such treatment. Conversely, the administration of high doses of vitamin C over a short duration may lead to pro-oxidative effects that could compromise the efficacy of antineoplastic therapies. This pro-oxidative mechanism is characterized by the generation of peroxides and ascorbyl radicals, which may significantly influence the tumor microenvironment and its progression [55,56].

Vitamin E, particularly α-tocopherol, is being investigated for its potential role as an adjunct in prostate cancer treatment, especially in the context of chemotherapy and radiotherapy. Studies suggest that α-tocopherol may modulate the growth of cancer cells, such as LNCaP prostate cancer cells, both under control conditions and during androgen stimulation [36,58]. The primary mechanism through which α-tocopherol influences prostate cancer progression is by reducing the concentration of the androgen receptor, which plays a key role in the development of this malignancy. The decrease in androgen receptor levels affects both the transcription process of this receptor and the translation of its protein, resulting in reduced activity [36,58].

Vitamin E exhibits differential effects on prostate cancer cells and prostate fibroblasts, inhibiting the proliferation of prostate cancer cells in a manner not directly related to its antioxidant activity. Although vitamin E possesses antioxidant properties, its impact on the expression and function of the androgen receptor is considered the primary mechanism through which it inhibits prostate cancer progression [36,58,59].

## 7. Balancing Benefits and Risks of Vitamins Intake in Prostate Cancer Prevention and Therapy

Numerous epidemiological and experimental studies have shown that deficiencies or excesses of certain vitamins—such as vitamins D, E, C, A and B vitamins, especially B12—can influence the initiation, promotion and progression of prostate cancer. Both the source of the vitamins (diet vs. supplementation) and the intake are crucial here, as a so-called biphasic effect—beneficial effects at physiological doses and potentially harmful effects at excessive intake—is observed for some components.

Vitamin E, like vitamin C, is a powerful antioxidant, but its effect on prostate cancer is highly controversial. The SELECT study showed that vitamin E supplementation at a dose of 400 IU per day was associated with a statistically significant increase in prostate cancer risk in healthy men [48]. A possible mechanism involves an imbalance between pro- and antioxidants—at very high doses, vitamin E may act pro-oxidatively, promoting cellular damage. Other studies indicate that the form of vitamin E (e.g., alpha-tocopherol vs gamma-tocopherol) may have different biological effects—some may even promote cancer progression with an unfavorable isomer ratio [60].

Although vitamin D has been shown to have anticancer effects, excess vitamin D can also be harmful. Vitamin D toxicity (hypercalcemia) can lead to kidney, heart and skeletal damage. In the context of prostate cancer, the data are inconclusive—some studies suggest that very high serum 25(OH)D concentrations (above 125–150 nmol/L, or 50–60 ng/mL) may be associated with an increased risk of certain cancers, although the mechanisms are not clearly confirmed [61,62]. It has been hypothesized that very high vitamin D concentrations may have an immunosuppressive effect or hormonal imbalance, which under certain conditions may promote cancer development. It is therefore advisable to monitor vitamin D blood levels during long-term supplementation, especially if doses exceed 4000 IU/day [63].

Vitamin B12 (cobalamin) plays an important role in DNA synthesis, methylation and cell division. However, over-supplementation of this vitamin, especially at doses well above the daily requirement (2.4 µg/day), may be associated with an increased risk of certain cancers, including prostate cancer. Some epidemiological studies have shown that high serum B12 levels (above 600–700 pmol/L) correlate with a higher risk of prostate cancer and more aggressive forms of the disease. The mechanism is not fully understood, but it is speculated that excess B12 may promote prostate cancer progression by enhancing cell division, promoting hypermethylation of oncogenic promoters and inhibiting suppressor genes [64]. A 2017 meta-analysis found that higher levels of vitamin B12 were statistically associated with a higher risk of prostate cancer, although not in all populations. Supplementation with high doses of B12 (e.g., 1000 µg or more) in individuals without deficiency should therefore be carried out with caution, especially in men over 50 [64].

Vitamin C is widely used in cancer prevention, mainly due to its antioxidant properties. However, in high doses (above 2000 mg per day), it can cause side effects such as diarrhea, gastrointestinal disorders and, in the long term, promote the formation of kidney stones (especially in men), which indirectly stresses the kidneys and can affect the patient’s overall health [65]. For prostate cancer, the use of intravenous megadoses of vitamin C (e.g., 10–50 g) aims to have a cytotoxic effect against tumor cells, but there is a lack of conclusive evidence for the efficacy of this clinical strategy, and the use of such therapies without medical control carries the risk of serious complications, including oxidative stress in healthy cells [66].

Vitamin A (retinol) has important functions in the regulation of epithelial cell differentiation and immune system function. However, in the context of cancer, including prostate cancer, its effects are ambiguous—it may exhibit both anti-cancer properties and potentially cancer-promoting properties when consumed excessively. The results of some studies suggest that a high intake of retinol, especially in the form of supplements, may be associated with a higher risk of prostate cancer, especially in its more aggressive forms. An NIH-AARP cohort study found that men with the highest serum retinol levels had a significantly higher risk of advanced prostate cancer, compared to men with lower levels [67]. The mechanism for this phenomenon may be related to the effect of excess vitamin A on prostate cell proliferation and reduced activity of certain suppressor genes. Excess retinol may also affect androgen metabolism, which in turn plays a key role in the pathogenesis of prostate cancer. On the other hand, moderate amounts of vitamin A and its precursor, beta-carotene, provided from the diet (e.g., carrots, pumpkin or yams) can promote protection against oxidative damage and support cell differentiation. The problem arises mainly with supplementation with high doses of retinol (more than 10,000 IU per day), which can disrupt cellular balance and promote tumorigenesis [68].

A balanced diet is fundamental in ensuring adequate intake of essential vitamins, thereby supporting overall health and physiological function. Such a diet typically provides sufficient quantities of most vitamins necessary for maintaining homeostasis and preventing deficiency-related conditions. However, when considering dietary supplementation, it is crucial to carefully evaluate and adjust the dosage to ensure safety and efficacy. This process should be based on evidence from clinical studies that establish optimal and safe supplementation levels, avoiding both deficiencies and potential toxicities associated with excessive intake. In clinical practice, assessing the need for additional vitamin supplementation involves a comprehensive evaluation of the patient’s dietary habits, medical history, and potential risk factors for deficiencies. Laboratory testing plays a vital role, as measuring serum levels of specific vitamins can provide objective data on an individual’s nutritional status. It is important to recognize that the daily diet naturally contains vitamins, and supplementation should be tailored accordingly. Unnecessary or excessive supplementation can lead to adverse effects, including hypervitaminosis. Therefore, clinicians should base supplementation recommendations on individual laboratory results, clinical signs, and evidence-based guidelines to ensure safety.

Further research is needed, as evidence suggests that the metabolism of individual vitamins varies significantly between individuals. Gaining a more comprehensive understanding of the factors that influence these metabolic pathways is key to improving therapeutic outcomes. Although tailoring vitamin supplementation can be resource-intensive, its potential benefits for people with prostate cancer and the broader healthcare system underscore its potential as a future research direction. Maintaining a balanced diet is the cornerstone of vitamin sufficiency, but targeted supplementation guided by laboratory assessments and clinical evidence is essential when dietary intake is insufficient or when specific deficiencies are identified. This approach optimizes health outcomes while minimizing risks associated with improper vitamin dosing.

## 8. Summary and Conclusions

A well-balanced diet, rich in vitamins and nutrients, may play a significant role in the prevention and therapy of prostate cancer. Vitamins A, D, E, C, and B-complex vitamins exhibit diverse mechanisms of action that can influence tumor development and progression.

The antioxidant properties of vitamins E and C help neutralize free radicals, potentially reducing the risk of DNA damage and mutations leading to carcinogenesis. Vitamin D is critical for the regulation of cell division and has been implicated in the inhibition of cancer cell proliferation. Furthermore, vitamin A is essential for cellular differentiation and may exert cancer-inhibiting effects. B-complex vitamins contribute to optimal cellular metabolism and may protect against oxidative stress.

Although numerous studies suggest potential benefits of vitamins in prostate cancer prevention, the findings are often inconsistent, and their effectiveness depends on multiple factors. Consequently, further clinical research is needed to elucidate the interactions between vitamins, their impact on different stages of the disease, and the potential adverse effects of their supplementation. It is particularly important to investigate whether high doses of certain vitamins, such as vitamin E or beta-carotene, may pose an increased cancer risk in specific patient populations. Table 3 provides an overview of the data for the specific vitamins discussed in this review.

## Figures and Tables

**Table 1:** Division of vitamins according to their solubility, role and occurrence.

Vitamin	Solu-bility	Storage Location	Role in the Body	Role in Cancer	Ref.
A (Retinol)	Fat	Liver, adipose tissue	Vision, immunity, skin and mucous membrane health	May have antineoplastic effects by regulating cell proliferation and differentiation, but excess intake may promote liver cancer	[13]
D (Cholecalciferol, Ergocalciferol)	Fat	Liver, adipose tissue	Regulation of calcium-phosphorus balance, bone health	May reduce the risk of certain cancers (e.g., colorectal) by regulating the cell cycle and inducing apoptosis	[10,11]
E (Tocopherol)	Fat	Adipose tissue, adrenal glands	Antioxidant, protects cell membranes	May have protective effects against cancer as an antioxidant, but high doses may increase the risk of prostate cancer	[13,15]
K (Phylloquinone, Menaquinone)	Fat	Liver	Blood clotting, bone metabolism	May have carcinopreventive effects by inhibiting cancer cell proliferation	[13]
B Vitamins (B1, B2, B3, B5, B6, B7, B9, B12)	Water	No significant location, small amounts in the liver and muscles	Metabolism of carbohydrates, lipids, and proteins, DNA synthesis, nervous and hematopoietic system function	May have protective effects against some cancers (e.g., colorectal, breast, esophageal), but excess intake of certain B vitamins (e.g., B9, B12) may promote cancer development and progression	[14]
C (Ascorbic Acid)	Water	No significant location	Antioxidant, collagen synthesis, immune function	May have protective effects as an antioxidant, but high doses may support the growth of certain cancers under specific conditions	[12,14]

**Table 2:** Comparison of Alpha-Tocopherol (α-T) and Gamma-Tocopherol (γ-T) [38,39].

Characteristic	Alpha-Tocopherol (α-T)	Gamma-Tocopherol (γ-T)
Level in the body	Predominates in human serum due to selective transport in the liver	Present in smaller amounts but also detectable in serum
Antioxidant activity	Strong antioxidant that protects cell membranes from oxidative stress	Potentially more effective in protecting against peroxynitrite-induced lipid oxidation
Impact on prostate cancer	Conflicting study results—supplementation in the SELECT trial increased prostate cancer risk	Potentially protective effect—inverse correlation with the risk of advanced prostate cancer
Anticarcinogenic mechanisms	Protects cells from oxidative stress but may disrupt the balance of other tocopherols	Induces apoptosis, inhibits cancer cell proliferation, and activates tumor-suppressing receptors (e.g., PPAR-γ)
Effect on tocopherol balance	High doses may reduce γ-T levels	Naturally present in the diet without negatively affecting α-T levels

**Table 3:** Summary of information on vitamins and prostate cancer.

	Main Mechanism of Action	Impact on Prostate Cancer	Role in Therapy	Concerns and Limitations	Positive Impact (dose)	Ref.
**Vit. A**	Regulation of proliferation, differentiation, and apoptosis through activation of RAR and RXR receptors	Inhibition of cancer cell proliferation and invasiveness, regulation of the cell cycle	May increase cell sensitivity to hormonal therapy and chemotherapy	Clinical trial results are inconclusive; further research is needed on safety	2500–5000 IU/day (from diet, e.g., beta-carotene)	[31,32]
**Vit. B**	Involvement in DNA synthesis, epigenetic regulation, transport via haptocorrin and transcobalamin	Conflicting results—low levels may increase risk, while supplementation may promote tumor progression	Impact on DNA methylation, which may modulate oncogene expression	Supplementation may increase cancer progression risk, while dietary folates may have protective effects	2.4 µg/day (from diet or moderate supplementation)	[64]
**Vit. C**	Antioxidant properties, reduction of oxidative stress and inflammation	May improve patients’ quality of life and support therapy	May increase cell sensitivity to hormonal therapy and chemotherapy	High doses may exhibit pro-oxidant effects and influence chemotherapy effectiveness	75–200 mg/day (from diet)	[45]
**Vit. D**	Regulation of cell proliferation and differentiation via activation of the VDR receptor	May slow cancer cell proliferation and enhance therapy effectiveness	May increase cell sensitivity to hormonal therapy and chemotherapy	No conclusive clinical evidence on supplementation efficacy; further studies are needed	800–2000 IU/day (maintenance of 25(OH)D: 50–125 nmol/L)	[46]
**Vit. E**	Modulation of androgen receptor levels, impact on cancer cell growth	May inhibit prostate cancer cell proliferation and reduce androgen receptor activity	May increase cell sensitivity to hormonal therapy and chemotherapy	Conflicting study results; in the SELECT trial, α-tocopherol supplementation increased prostate cancer risk	<100 IU/day (from diet, gamma-tocopherol)	[37]

## Data Availability

No new data were created or analyzed in this study.
